# Graph Theoretical Model of a Sensorimotor Connectome in Zebrafish

**DOI:** 10.1371/journal.pone.0037292

**Published:** 2012-05-18

**Authors:** Michael Stobb, Joshua M. Peterson, Borbala Mazzag, Ethan Gahtan

**Affiliations:** 1 Department of Mathematics, Humboldt State University, Arcata, California, United States of America; 2 Department of Psychology, Humboldt State University, Arcata, California, United States of America; 3 Department of Biology, Humboldt State University, Arcata, California, United States of America; Wake Forest School of Medicine, United States of America

## Abstract

Mapping the detailed connectivity patterns (connectomes) of neural circuits is a central goal of neuroscience. The best quantitative approach to analyzing connectome data is still unclear but graph theory has been used with success. We present a graph theoretical model of the posterior lateral line sensorimotor pathway in zebrafish. The model includes 2,616 neurons and 167,114 synaptic connections. Model neurons represent known cell types in zebrafish larvae, and connections were set stochastically following rules based on biological literature. Thus, our model is a uniquely detailed computational representation of a vertebrate connectome. The connectome has low overall connection density, with 2.45% of all possible connections, a value within the physiological range. We used graph theoretical tools to compare the zebrafish connectome graph to small-world, random and structured random graphs of the same size. For each type of graph, 100 randomly generated instantiations were considered. Degree distribution (the number of connections per neuron) varied more in the zebrafish graph than in same size graphs with less biological detail. There was high local clustering and a short average path length between nodes, implying a small-world structure similar to other neural connectomes and complex networks. The graph was found not to be scale-free, in agreement with some other neural connectomes. An experimental lesion was performed that targeted three model brain neurons, including the Mauthner neuron, known to control fast escape turns. The lesion decreased the number of short paths between sensory and motor neurons analogous to the behavioral effects of the same lesion in zebrafish. This model is expandable and can be used to organize and interpret a growing database of information on the zebrafish connectome.

## Introduction

### Individually identified neuron connectomes

Studies of individually identifiable neurons have a long history in neuroscience [1]. One advantage to studying identified neurons is that ‘the same’ neuron can be studied in different animals, allowing a rich database of structural and physiological information about a neuron to be compiled. Identified neurons have been useful for studying neural circuit structure at the cellular level, where the goal is a precise map of all of the synaptic interactions among neurons in a functional circuit [2]. This approach has proven feasible in invertebrate models, such as Drosophila [3] and C. elegans [4], with many identifiable neurons. However, the size of such datasets, even for relatively simple circuits, poses challenges for interpreting connectome data. Thus, computational representations of connectomes are necessary for meaningful analysis. Graph theory is an increasingly popular computational framework for analyzing connectome data. Graph analysis can be applied to neural circuits at different spatial levels, including identified neuron networks.

**Figure 1:**
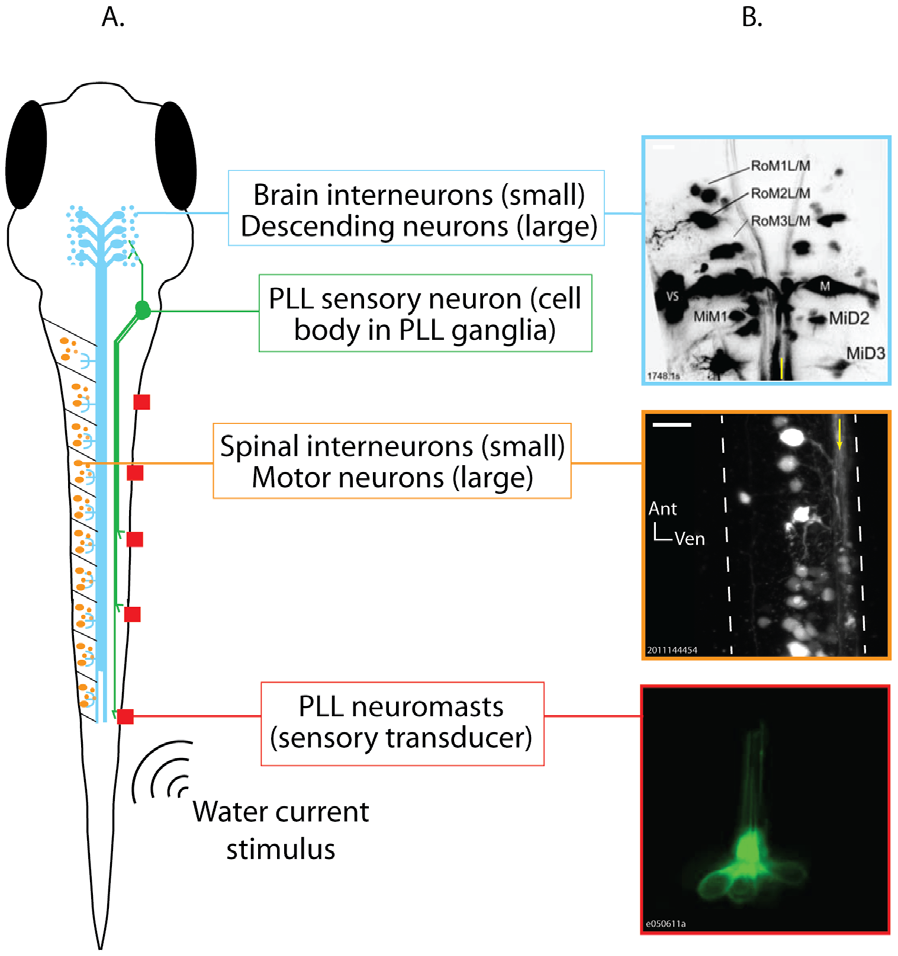
The posterior lateral line (PLL) sensorimotor pathway in zebrafish larvae. A. Schematic representation of a 6 day old zebrafish larva (dorsal view) showing, in different colors, all of the neuron types included in the model pathway, and the effective stimulus for hair cell activation (water current). Neuron types are labeled to the right of the schematic. B. Photomicrographs showing individually identifiable neurons within the PLL sensorimotor pathway. Top: Descending neurons in the hindbrain of a 6 day old zebrafish larva after injection labeling with a fluorescent tracer. The Mauthner neurons (M), Mauthner segmental homologs (Mid2, MiD3), and other identifiable descending neurons can be seen. The image is a maximum projection of 22 optical sections taken at 3 µm intervals through the dorsal-ventral axis of the hindbrain. Anterior is up and the yellow line shows the approximate midline. Middle: Spinal neurons imaged in a 7 day old larva prepared as described for A. The image is a maximum projection of 10 optical sections taken at 2 µm intervals through the rostral spinal cord at approximately the level of the 8^th^ myotome. The dashed white lines trace the spinal cord's boundaries, and the yellow arrow is drawn along the bundle of descending axons running down the ventral spinal cord. Bottom: A PLL neuromast. This neuromast was imaged in a single optical section in a 6 day old Brn3c:eGFP transgenic larva, in which all lateral line neuromasts express GFP. Individual hairs (cilia) can be seen extending toward the top of the image. Sensory neurons (not shown) contact the hair cell bodies, which can be seen at the bottom of the image. Scale bars = 20 µm.

We used graph analysis to study a sensorimotor neural pathway in the zebrafish larvae that is largely comprised of individually identifiable neurons (Figure 1). The pathway arises from the posterior lateral line (PLL), a sensory system present in most fish that consists of mechanosensory hair cell clusters (neuromasts) that are distributed on the body surface. Lateral line neuromasts detect water current and are important for many behaviors in fish, including schooling, detection of prey and predators, object localization, and rheotaxis [5]–[7]. Ascending sensory neurons carry PLL signals to the hindbrain, a major sensorimotor integration area. Hindbrain neurons that descend to the spinal cord relay processed PLL signals to spinal motor networks, and the pathway ends with spinal motor neuron output to muscles. This pathway underlies the ability of fish to make adaptive locomotor responses to PLL stimulation.

There have been numerous studies of the PLL in zebrafish and of downstream sensory and motor neurons involved in PLL-mediated behaviors. Many of these neurons are individually identifiable in larvae, a feature that distinguishes this connectome from other vertebrate connectome models. Posterior lateral line hair cells and corresponding sensory neurons are identifiable by their location on the skin surface [6], [7]. Descending neurons in the brain, which number approximately 300, are identified by their somatic and dendritic morphologies and positions within hindbrain rhombomeres [8], [9]. Spinal cord neurons, of which there are at least 10 types, are identifiable by their morphologies and the spinal segments in which they reside [10]–[12]. The same studies have also shown that these neurons have highly consistent anatomical and physiological properties across individual animals, which is essential for investigating general principles of the connectome's structure.

### Graph analysis of neural connectomes

Graph theory has been successfully applied to the problem of analyzing connectomes [13]–[15]. All networked systems have a topology, and graph theory provides tools to analyze network topologies to understand how they constrain network functions. Graph representations of neural connectomes are comprised of nodes (vertices), representing individual neurons or brain areas, and connections (edges) that link nodes. The structure of a graph can be described with mathematical metrics that reflect functional properties of the connectome. Although many metrics can be defined and computed, the current analysis focuses on two functional network properties, 1. efficiency of signal propagation through the network, and 2. resilience of signal propagation to localized network injury. These properties were assessed by comparing the graph analytic metrics from the zebrafish network to those of ‘test networks’ with known structural and functional properties. This initial analysis determined whether the properties of the zebrafish PLL graph fell within expected ranges on computational and biological parameters.

**Figure 2:**
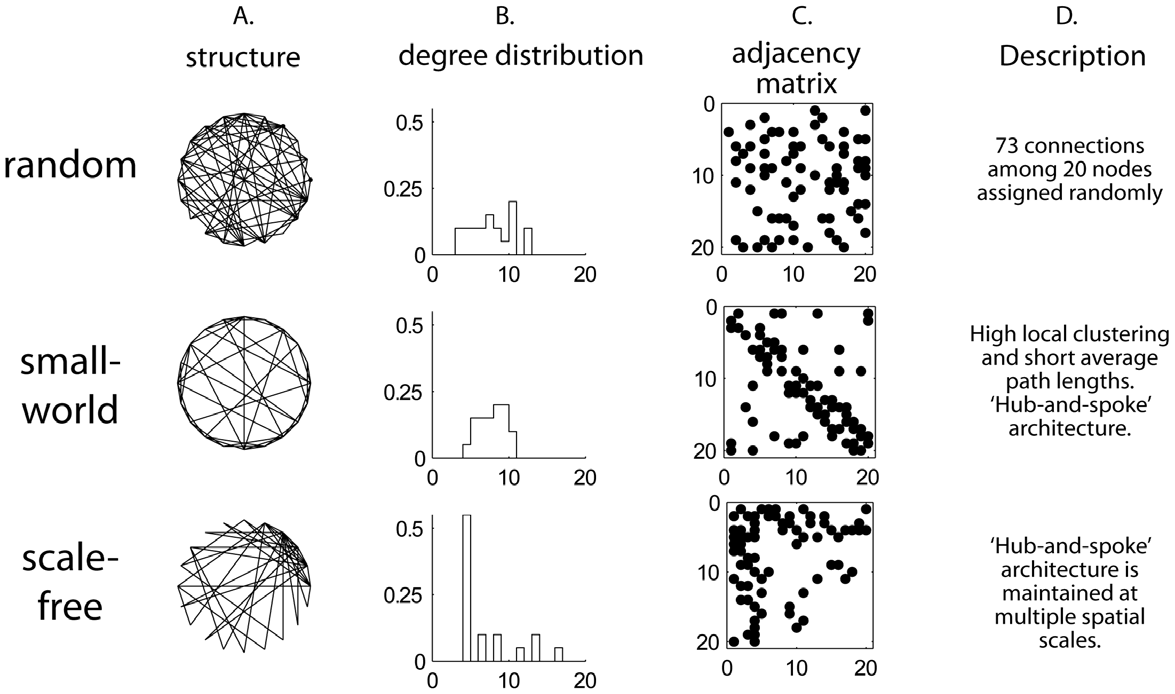
Properties of random and small-world graphs. Random, small-world and scale-free networks containing 20 nodes and 73 connections were generated by computer algorithms. The number of nodes and connections were chosen to be small to highlight differences between graphs. A. A structural representation of each graph. The nodes are arranged in a ring and connections are lines drawn between the nodes. The sparser appearance of the small-world graph occurs because most connections are between nearest neighbors along the perimeter of the ring, whereas in the random graph, short and long distance connections are equally probable. The scale-free graph has a hub in the upper right portion of the graph. B. Degree distribution. In random graphs, degrees are roughly normally distributed. In the small-world network, the degree distribution has a smaller standard deviation relative to random graphs because most nodes have a similar connectivity pattern. The scale-free graph has a “power-law” (approximately exponential) degree distribution. C. Adjacency matrices. Both axes show node number, and connections are represented as dots in the matrix. There is a uniform distribution of connections in the random graph. The thick diagonal band in the small-world distribution results from the high frequency of connections between nodes in neighboring positions on the ring. The thick horizontal and vertical bands in the adjacency matrix for the scale-free network correspond to hubs. D. Description of connectivity patterns.

‘Small-worldness’ is the graph metric that was used to assess the efficiency of signal propagation in the modeled pathway. The term ‘small-world’ network was coined by Watts and Strogatz [16] and has been since recognized as an important emergent property of a wide range of complex networks. Small-worldness is based on two structural features of a graph, the clustering coefficient and average path length. Clustering occurs when ‘neighbor’ nodes that are connected to a particular target node are also likely to be connected to each other. Average path length refers to the mean number of edges that must be traversed to travel between any two randomly selected nodes on the graph. Small-worldness occurs when, in sparsely connected networks, clustering is high and the average path length between nodes is short (Figure 2). Small-worldness has been demonstrated for neural networks in the cat hindbrain [17] and in several cortical networks [18], [19]. Small-world organization in neural systems allows incoming signals to be processed in areas of high local clustering and output to be sent efficiently (with few steps) to downstream circuits or effectors [16], [20].

Resilience to localized network injury was assessed in two ways. First, the degree distribution of the zebrafish connectome was compared to that of ‘scale-free’ test graphs. Degree distribution is a probability distribution function that gives the likelihood that an arbitrary node has a given number of connections (Figure 2). While random networks have a Gaussian degree distribution, indicating that most nodes have an average number of connections, scale-free graphs have an exponential (or “power-law”) degree distribution with a few very highly connected nodes and many more sparsely connected nodes. Scale-free networks have been shown to be resilient to random attack as compared to random networks, but more vulnerable to targeted attack on highly connected ‘hub’ nodes [21].

The second method for assessing resilience to attack was to delete nodes in the graph and to examine the effects on graph metrics. Specific nodes were targeted for deletion, corresponding to identified zebrafish neurons involved in fast escape behaviors. The extent to which node deletions disrupted signal propagation through the pathway was determined by analyzing paths lengths between neuromasts to spinal motor neurons. Paths of length 3 are the most direct connections possible between neuromasts and motor neurons in the model and represented the fastest sensorimotor responses. Only paths of length 3 and 4 were analyzed because short paths were considered most relevant to the fast escape responses being modeled.

## Methods

### Ethics Statement

All studies involving zebrafish larvae were done in accordance with an approved protocol issued by the Humboldt State University animal care and use committee to Dr. Ethan Gahtan (protocol number 08/09.P.45.A)

### Biological basis of the of the zebrafish sensorimotor pathway model

Most of the information about zebrafish neurobiology that was used in the model was gleaned from published scientific research (Table 1). In addition, neuromasts, hindbrain neurons, and spinal neurons were imaged in larval zebrafish to illustrate structures and, in the case of neuromasts, to perform cell counts (Figure S1).

**Table 1:** Rules governing distribution of connections in the zebrafish model network.

Connection from	Connection to	Rules
Sensory	Sensory	A. 35 neuromast-to-sensory neuron connections per side [19]. B. Ipsilateral connections only [7]. C. Each neuromast connects to at least one sensory cell [22]. D. Each sensory cell connects to at most 5 (adjacent) neuromasts. E. Number of neuromasts a sensory neuron connects to is distributed exponentially [19]. F. Total of 70 connections.
Sensory	Brain	A. Each sensory neuron connects to 25 brain neurons; All connections are ipsilateral [22]. B. Sensory neurons only project to neighboring brain segments. C. Sensory projections are somatotopic such that more caudal neuromasts connect (via sensory neurons) to more caudal brain segments [7], [54]. D. Most caudal and rostral neuromasts each connect to two brain segments, with 80% of connections to the most caudal and rostral brain segment, respectively. E. Mid-body neuromasts connect to three brain segments each, with the most connections (40%) to the middle brain segment. F. Mauthner neuron has direct connections from the PLL [55]. G. Total of 999 connections
Brain	Brain	A. Hindbrain neurons are arranged in 7 segments [56]. B. Connections between brain interneurons in same or adjacent segment only. C. Descending neurons with dendrites in another hindbrain segment [8] receive connections from that segment via a brain interneuron. D. Brain interneurons make ascending and descending connections in adjacent segments on both sides of the brain, or have more restricted connections [24]. E. Descending neurons receive more of their connections from interneurons within their own hindbrain segment (75% in the model) [57]. F. Descending neurons CRN, MiT, RoM2M, RoM2L, RoM3 connect to other descending neurons with 1,750 connections (<1% of total) [8], [54]. G. Total of 1,750 descending-descending neuron connections, 32,535 descending-interneuron connections, and 35,700 interneuron-interneuron connections.
Brain	Motor	A. Each descending neuron makes 10 connections per spinal hemisegment in all hemisegments its axon reaches. B. Mauthner series cells descend to the caudal most spinal segment and connect to motor neurons in each segment [58] with a 99.6% probability in the model. C. Total of 34,800 connections (18,045 descending-spinal interneuron connections and 16,755 descending-spinal motor neurons connections). D. Length (number of segments) and direction (ipsilateral, contralateral, or bilateral) of descending axon projections were assigned as follows [25], [54]: CRN, CC, and IC all descend 5 segments; RoR1, RoL2; RoL2c, and MiD2i descend 8 segments; MeM, MeM1, MeL, MeLr-m, MeLc, RoM1r, RoL1, Vestibulospinal descend 13 segments; RoM1c, RoM2m, RoL2r, RoV3, RoL3, MiD2cm, Mid3cm-cl, MiD3i, MiT descend 18 segments; RoM2l, RoM3m-l, Mauthner, MiM1, MiV1, MiR1, MiR2, MiV2, MiD2cl, CaD, CaV descend 25 segments; Mauthner, MiD2c, MiD3cm, MiD3cl, CaD, CRN made contralateral connections; 84.8% of descending neurons connect to ipsilateral cord.
Motor	Motor	A. Each spinal hemisegment had 14 interneurons [11]. B. Each spinal hemisegment has 3 primary and 10 secondary motor neurons [10]. C. 25% of a spinal interneuron's connections are within its own segment. D. Spinal interneurons have a 29.2% probability of connecting to other spinal interneurons, and a 72.8% probability of connecting to motor neurons. E. Spinal motor neurons were not connected to other neurons. F. Total of 61,244 connections (17,892 spinal interneuron-spinal interneurons connections and 43,352 spinal interneuron-spinal motor neuron connections). G. There were 8 types of spinal interneurons. Their names, numbers (per hemisegments), and properties were as follows [11]: CoPA, 1, ascends contralaterally to segment 1; CoSA, 2, ascends contrallaterally10 segments; CoLA, 1, ascends contrallaterally 5 segments; CiD, 2, descends ipsilaterally 13 segments; MCoD, 2, descends contrallaterally13 segments; UCoD, 2, descends contrallaterally 14 segments; VeMe, 2, descends Ipsilaterally 9 segments; CoBL, 2, ascends and descends contralaterally 4 segments

**Figure 3:**
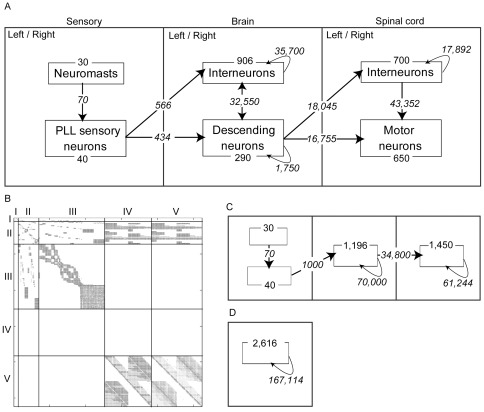
Graph structures, showing the numbers of nodes and connections. The outer squares represent processing compartments. Nodes are shown as rectangles within processing compartments, and node numbers are shown along the borders of those rectangles.Connections (edges) are shown as arrows, and connection numbers are shown near the respective arrows. The total number of nodes and connections is the same for all graphs. A. The zebrafish connectome graph model. This schematic does not show the many individual cell-to-cell connection rules that further distinguished the zebrafish graph. Left and right sides of the nervous system are shown together here to aid visualization, but in the actual graph they were distinct, and some connections were ipsilateral and others contralateral (see Table 1). B. Adjacency matrix for the zebrafish graph. Connections are from nodes in rows to nodes in columns. I = neuromasts and sensory neurons, II = descending neurons, III = brain interneurons, IV = spinal motor neurons, V = spinal interneurons. Spinal motor neuron nodes made no connections to other nodes so are not shown in the matrix. C. Structured random graph model. This test graph was the most similar to the zebrafish graph but did not have bilaterality or distinct node categories in brain or motor compartments. All permitted connections in this model were assigned randomly. D. The random graph. Any connection was permitted and all connections were assigned randomly.

Rules not associated with citation numbers were estimations made by the authors. The number of each type of neuron and connection in the model is given in Figure 3.

Confident estimates could be made of the types of cells involved in the pathway and the numbers of each type in ∼6 day old zebrafish larvae (Figure 3). In total there were 2,616 model neurons and 167,114 connections. Many specific connections among these neurons have also been described and were incorporated into the model, but most connections were assigned according to stochastic rules. The zebrafish network consisted of three primary compartments– sensory, brain, and spinal cord– and connections within and between compartments (Figure 3). This division reflects the general organization of vertebrate sensorimotor reflex pathways. Each compartment in the model contains two types of interconnected nodes, modeling relevant features of the neurobiology. Table 1 presents the connection rules that guided construction of the model, along with citations for specific rules.

**Table 2:** Small-world index, Clustering Coefficient, and path length averaged across 100 instantiations for each graph model.

Graph Type	Small-world index	Clustering Coefficient	Characteristic Path Length
zebrafish PLL connectome	4.13	0.1323	2.8643
small-world	19.83	0.6141	2.7658
structured random	1.66	0.0399	2.1530
Random	1.00	0.0244	2.1796

### Sensory compartment

Two types of sensory nodes were included: neuromasts, which are the sensory transducers, and primary sensory neurons, which relay signals from neuromasts to the brain. PLL neuromasts occur in two stripes along the side of the fish's body. There are approximately 8 PLL neuromasts per side in a 6 day old zebrafish larva [22] (Figure S1). Each neuromast is composed of 8 hair cells, half of which respond best to water flow in the anterior direction and half to water flow in the posterior direction [7], [23]. The model does not represent individual hair cells. Instead, each neuromast is represented by two nodes (simply called ‘neuromasts’ in the model) to account for the two sets of directionally tuned hair cells, and as a result the model contains double the number of PLL neuromasts usually found in a 6 day old larva. Usually, two sensory neurons contact each neuromast [19]. In total, the model contained 30 neuromasts and 40 sensory neurons. Sensory neurons relayed signals from neuromasts to the ipsilateral hindbrain, with different sensory neurons having outputs targeted to different hindbrain segments.

### Brain compartment

Neurons in the hindbrain form sensorimotor processing circuits that receive sensory signals and transform them into motor signals. Nearly all sensory systems connect to the hindbrain in zebrafish, and hindbrain circuits mediate many types of sensorimotor reflexes that allow the fish to interact adaptively with its environment [24]. The hindbrain is organized into 7 distinct segments (called rhombomeres) distinguished by gene expression patterns and by the morphological and physiological properties of the neurons they contain [25]. Hindbrain segments in zebrafish are named according to their position along the rostral-caudal axis, with Ro1-Ro3 corresponding to the rostral 3 segments, Mi1-Mi3 to the 3 middle segments, and Ca1 to the caudal-most segment [26]. The model hindbrain was also divided into 7 segments. This was an important feature because several connection rules favored connections between adjacent segments (see Table 1).

Descending neurons are one distinct group of hindbrain neurons. They project long axons into the spinal cord (and, in some cases, also into the brain) and they form the main pathway linking the brain to the spinal cord (Figure 1). Descending neurons have been studied extensively in larval zebrafish. Most are individually identifiable and have been assigned unique names based on their morphologies, positions within the hindbrain, and axonal projection patterns [8]. This naming system has been preserved in the model. The model included 145 descending neurons per side (290 total), which is consistent with previous studies in larval zebrafish [8], [27]. When information was available about the synaptic connections of individual descending neurons, we incorporated that into the model. This included features such as the length and direction of axon projections (Table 1). The Mauthner neuron is a large descending neuron present in zebrafish and in other fish and amphibian species. The Mauthner neuron, along with its two segmental homologs (together, the ‘Mauthner series’) control fast escape turns. Information about the synaptic connectivity of Mauthner series neurons was included in the model, and information about their function in escapes motivated a ‘simulated lesion’ in which the Mauthner series nodes were deleted from the model.

There are also many local interneurons contained within the hindbrain which form connections with sensory and descending neurons. Interneuron density was estimated by counting hindbrain cell nuclei in digital images of methylene blue stained brain sections of 120 hour old zebrafish larvae. The images were downloaded from an online zebrafish anatomy atlas (available at www.zfin.org) and are of sufficient quality and resolution (>5 megapixels) to unambiguously count cell nuclei. Cell densities were sampled from three, 50 µm^2^ areas of each image within the brain region where descending neurons occur (a narrow band running the length of the hindbrain and into the midbrain). The three sampled areas were selected by visual inspection to be areas of high, low, and intermediate density of cell nuclei, and all stained nuclei within each section were counted. Average density across samples was scaled up to account for the full area of the sensorimotor hindbrain, yielding an estimate of 19,500 interneurons (Figure S2). This number did not account for areas of neuropil in the hindbrain or nuclei from non-neuronal cells. Moreover, even among hindbrain interneurons, only a subset would be expected to contribute to PLL processing. We ultimately included 906 interneurons, assigning 3 interneurons to each descending neuron, and an additional 36 interneurons in hindbrain regions in the model where no descending neurons occurred. A range of morphological types of hindbrain interneurons have been found, including neurons with both ascending and descending axons to both sides of the hindbrain, and others with more restricted projection patterns [24]. All of these reported projection patterns were included in the model.

### Spinal cord compartment

The vertebrate spinal cord contains complex motor networks, controlled by descending signals from the brain, that generate precise motor outputs to muscles. In fish, a basic motor circuit, consisting of motor neurons and interneurons, repeats in each segment of the spinal cord. There are ∼25 spinal segments in zebrafish larvae, each with symmetrical left and right sides (hemisegments). There are at least 10 distinct types of neurons present in each spinal cord segment in larvae, including 2 types of motor neurons (primary and secondary) and 8 types of interneurons [11]. The model included all of these neuron types in the numbers they occur in zebrafish larvae and also incorporated known differences in their axonal projection patterns.

Following the rules summarized in Table 1, 100 instantiations of the connectome graph were created. In these graphs, large scale connection patterns remained the same, though all individual connections were stochastically generated. The adjacency matrix for each graph was stored, and subsequent analyses were done on these 100 instantiations of the connectome.

### Test networks

In addition to the zebrafish model network, we generated 3 types of test networks: 1. Structured random, 2. Random, and 3. Small-world (Figures 2 and 3). The zebrafish network was compared to these test networks to assess how the biologically-based connectivity patterns affected graph properties. To isolate the effects of connectivity, all of the test networks were given the same number of nodes and edges and differed only in how connections were distributed. One-hundred instantiations of each test network were created and stored for analysis.

The small-world network was generated using a standard, published algorithm [16]. In the small-word network, 94% of connections were to nearest neighbors, with 6% randomly assigned. The structured random network, coded by the authors, maintained the same 3-compartment organization as the biological model, and the same number of connections between compartments. All specific connections in the structured random model were assigned randomly. Existing Matlab codes were used to generate the random network [28]. In the random network, connections between nodes were assigned according to a uniform, random distribution and there was no compartmental structure to the network.

### Definitions of graph theoretical measures

The Brain Connectivity Toolbox for Matlab was used to compute graph metrics [28]. We used the average across all 100 instantiations to compare the different networks on each of the graph metrics. The method for computing graph metrics is described in detail by Sporns et al. (2011) [28]. Degree distribution is defined as the probability P(k) associated with the graph that gives the likelihood that a randomly selected node has degree k. Small-worldness (S) was computed according to the method described by Walsh (1999), specifically, S = γ/λ, where γ is the ratio of the clustering coefficient of the target graph to the clustering coefficient of a random graph of the same size, and λ equals the ratio of the average path length of the target graph to the average path length of a random graph of the same size [29]. Path lengths were calculated using a function in the Brain Connectivity Toolbox. This function allows nodes and path lengths of interest to be selected, and the software returns the number of all distinct paths of the given length between nodes. Only paths of length 3 and 4, emanating from neuromasts and leading to motor neurons, were examined.

A modularity analysis was performed on anatomically-constrained graphs using a community detection algorithm within the Brain Connectivity Toolbox [28]. The community vector C identifies groups of nodes (modules) that are more strongly connected to each other than to nodes outside the group. As an additional criterion, a node had to belong to the same module across all 100 instantiations of the graph (by finding the intersection of the modules) or was dropped from the module. Only the descending modules are shown, but these modules are strongly influenced by sensory inputs and spinal outputs shared by descending neurons. To measure the participation of nodes from the sensory, brain and spinal compartments, the average number of each type of node within modules, across all instantiations of the graph, was calculated.

## Results

Our connectome model of the PLL sensorimotor pathway in zebrafish larvae consisted of 2,616 model neurons with 167,114 connections. The total connection density was 2.45% of all possible connections. Four properties of the zebrafish graph were analyzed: small-worldness, scale-freeness, modularity, and the effects of targeted node deletion on path length frequency.

### Small-worldness

Table 2 shows the small-world index for each graph type analyzed. The zebrafish graph had an average small-world index, across 100 instantiations of the graph, of 4.13±0.01, significantly higher than the structured random network (p<.001) or the random network (p<.001). A graph that was designed to maximize small-worldness, with the same number of nodes and connection density, had a small-world index of 19.83±0.06.

### Scale-freeness

**Figure 4:**
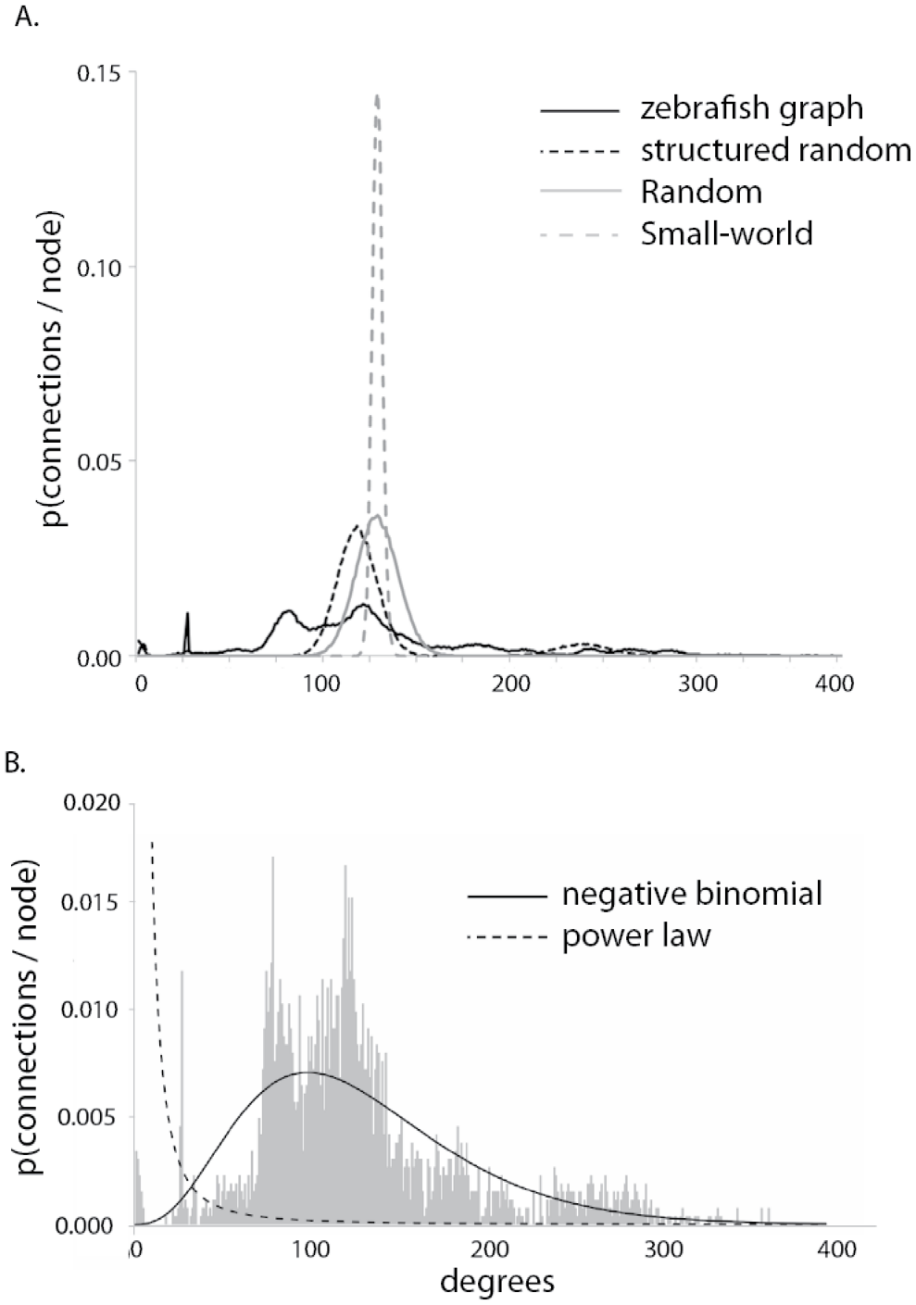
Degree distribution. Degree distribution depicts the probability of nodes with a given degree occurring in the graph. The zebrafish model and 3 test models had the same overall connection density but a different distribution of connections, as indicated in the chart. The zebrafish model had the broadest range of degrees.

The property of scale-freeness is defined to be a degree distribution in which the degrees have a power-law distribution. Graphs with a degree distribution best fit by a power law distribution are called scale-free. There was a wider range of degrees in the zebrafish graph than in any of the test graphs (Figure 4A). Most nodes (∼80%) had a degree between 75 and 150, but the zebrafish graph had nodes with much higher degrees (>400) than in any of the test networks. There was also a peak at ∼25 degrees, primarily due to the presence of 40 sensory cells that made relatively few connections each. Among the test graphs, the structured random model was most similar to the zebrafish network, but it still lacked nodes with as many degrees as the zebrafish graph. Average number of degrees for nodes in the small-world network was approximately 130, with relatively low variability (128.76±5.44) around this peak. Akaike's Information Criterion (AIC) was used to examine the relative fit of various models to the degree distribution of the zebrafish graph. The relative goodness of fit for these model distributions, from best to worst, was as follows: Negative binomial, Gamma, Weibull, Normal, Exponential, Geometric, Power-law, Poisson. Although there was a large range of degrees in the zebrafish graph, the Power-law distribution was one of the worst fit distributions (ΔAIC essentially zero), so the network was not scale-free (Figure 4B).

Table 3 shows degree distribution among identified descending neuron types in the model, placed into three broad categories. Several types of descending neurons have a very large number of connections (>200), with a maximum of approximately 400. These very high-degree nodes include cranial relay neurons (also called T-cells; Table 2; Figure S3), IC, CC and RoM1 cells.

### Modularity Analysis

**Figure 5:**
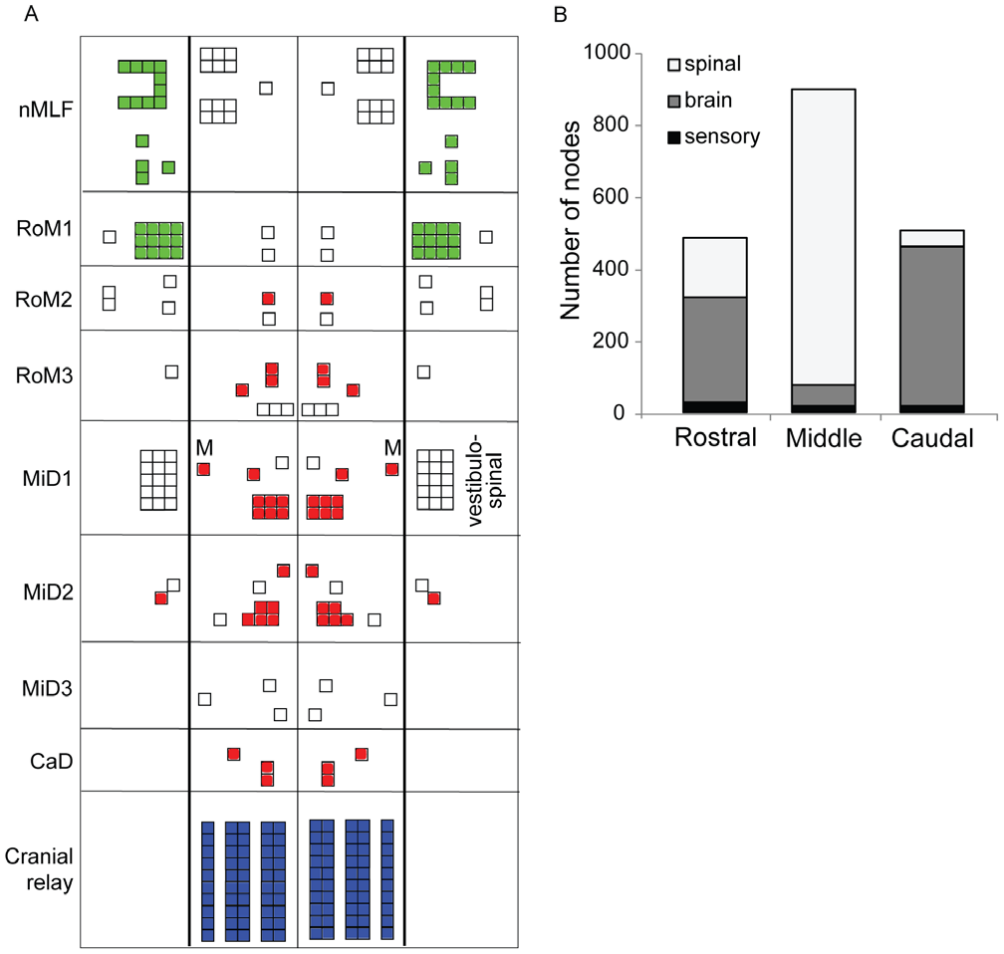
Network modularity. Modules of high interconnectivity were identified using a community detection algorithm. A. A schematic of the zebrafish descending neuron population showing the 3 modules detected in separate colors. Only nodes that placed in the same module across all 100 instantiations of the network were included. Distinct rostral (green), middle (red), and caudal (blue) groups are apparent. Major segments of the hindbrain are labeled for anatomical reference, as are the midbrain nMLF, Mauthner neurons (M), and vestibulospinal neurons. B. The average number of nodes in each brain module (rostral, middle, caudal) across 100 instantiations of the network is shown by the height of the bars. The number of sensory, brain, and spinal nodes within each module is shown by the shading. Nodes in each module largely share afferent and efferent connectivity.

The average number of modules identified across 100 instantiation of the network was 6.38±2.46. Three distinct modules were identified among descending nodes after intersecting modules across all instantiations of the network. These 3 modules roughly align with rostral, middle, and caudal sections of the hindbrain (Figure 5A). Very few sensory and spinal nodes belonged to the intersection because different nodes were included in each instantiation. The average number of nodes, without intersecting modules, was 483.66±14.34 in the rostral group, 894.66±7.73 in the middle group and 503.65±3.72 in the caudal group. Sensory nodes contributed equally to each module, but the proportions of brain and spinal nodes varied across the modules (Figure 5B). Modularity among descending nodes was primarily determined by the shared sensory inputs and spinal outputs contained within each module.

**Table 3:** Distribution of degrees among identified descending neuron types in the zebrafish model.

	Degree>200	75<Degree<150	Degree<50
Model identified neuron	T-cells, IC, CC, RoM1	MeL, MeLR, MeLM, MeLC, MeM, MeM1, RoR1, vestibular spinal, Mauther, MiR1, MiR2	RoL1, RoM1R,RoL2, RoL2, RoL2R, RoL2C, RoM2L, RoM2M, RoM3M, RoM3L, RoV3, MiM1, MiV1, Mid2CM, Mid2CL, MiT, Mid21, MiV2, Cad, Cav
Number of nodes	102	92	96

### Targeted node deletions

**Figure 6:**
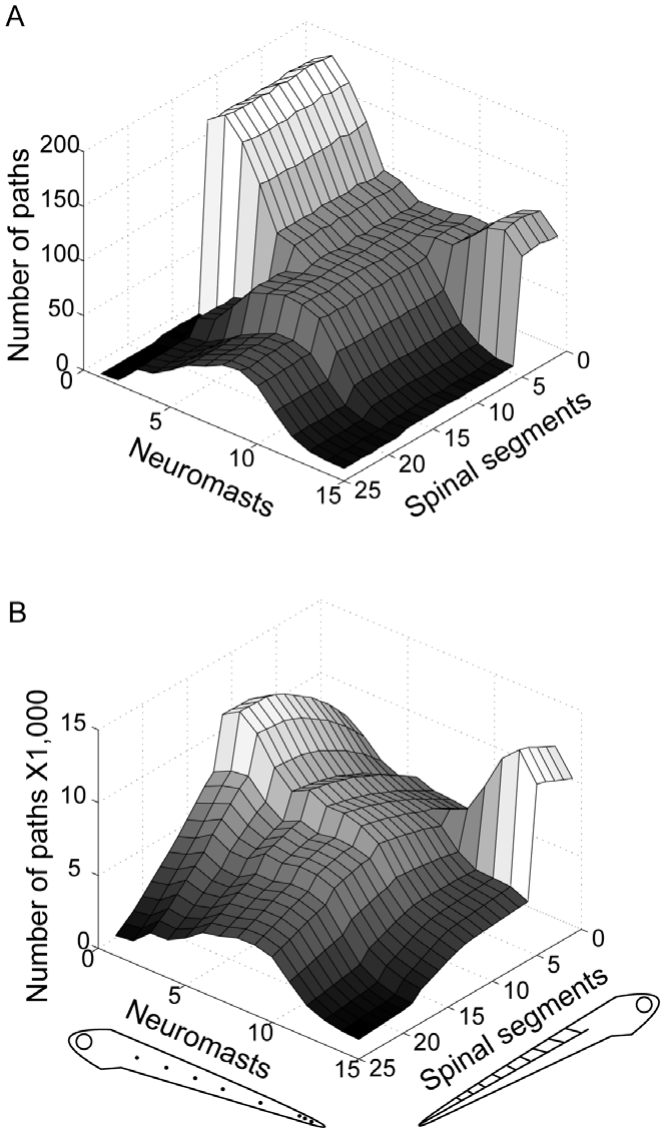
Path lengths between neuromasts and spinal motor neurons. Number of paths of length 3 (A) or 4 (B) between each neuromast and motor neurons in each spinal segment for the zebrafish model. Each point on the graph represents the number of distinct paths connecting a given neuromast and spinal segment. Two paths are considered distinct if they have at least one edge that is not shared. Larval zebrafish silhouettes at the bottom show the orientation of axes where neuromast and spinal segment numbers are plotted, with higher number corresponding to more caudal locations (also applies to Figure 7).

Deleting specific nodes from the graph altered the number of short paths of length 3 or 4 connecting neuromasts and motor neurons. The intact model had an average of 70.80±0.37 paths of length 3, and 6,534.60±25.50 paths of length 4, between neuromasts and spinal segments. The number of shortest paths (paths of length 3) between neuromasts and rostral spinal segments was greatest for rostral and caudal neuromasts as compared to mid-body neuromasts. In contrast, the number of shortest paths between neuromasts and caudal spinal segments was greatest for mid-body neuromasts (Figure 6).

The number of paths of length 3 were different for neuromasts at different locations along the body, but trend was attenuated for paths of length 4 (Figure 6). There is also a qualitative difference in the connectivity patterns of these different path lengths. For paths originating at mid-body neuromasts (5–10), the number of paths of length 3 consistently decreases from rostral to caudal spinal segments. In contrast, for paths of length 4, there is a peak at about the 5^th^ spinal segment. This means that although the number of shortest-length (3-step) connections decreases for more caudal parts of the spinal cord, this isn't necessarily the case for more indirect paths.

**Figure 7:**
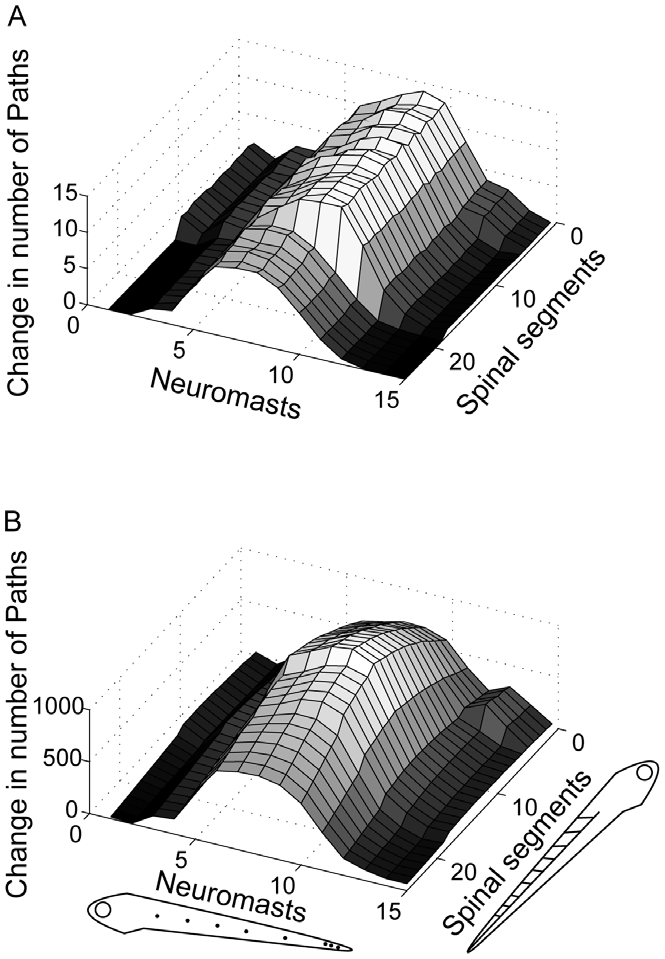
Mauthner series deletion. The effect of deleting the 6 model Mauthner series neurons (3 on each side) on path lengths between neuromasts and spinal motor neurons was examined separately for paths of length 3 (A) and length 4 (B). As in Figure 6, higher numbers on the two bottom axes represent more caudal positions on the larva's body.

Deleting the Mauthner series reduced the number of paths of length 3 and 4 between neuromasts and spinal motor neurons (Figure 7). In the deleted model, the average number of paths was 65.08±0.36 and 6,048.16±23.90 for paths of length 3 and 4, respectively, which is a reduction of 8.08% and 7.44%. Therefore, shorter paths were somewhat more vulnerable to the effects of Mauthner series deletion. The loss was not even across somatotopic locations and was different between the two path lengths at different locations. For both path lengths, paths originating at mid-body neuromasts were most affected. This can be explained by the fact that mid-body neuromasts were more likely to make connections to middle hindbrain segments, where the Mauthner series neurons were located. When considering paths of length 3, paths ending in rostral spinal cord were more affected than paths ending in middle or caudal spinal cord, but that trend was not present when considering paths of length 4, which decreased more evenly along the spinal cord.

A control deletion experiment was done in which an equal number of nodes was selected randomly for deletion from within the same hindbrain segments occupied by the Mauthner series. The selected nodes were a vestibulospinal, an MiV2, and an MiD3cl node. Each was deleted bilaterally. One-hundred instantiations of the random deletion network were analyzed as described for the Mauthner series deletion. Random deletions resulted in a 6.25% reduction in the number of 3-step paths and a 5.57% reduction in 4-step paths between neuromasts and motor neurons relative to the intact model. This was a significantly smaller effect on path length reduction than resulted from Mauthner series deletion for both 3-step (t(99) = 14.90, p<0.001) and 4-step (t(99) = 20.37, p<0.001) paths.

## Discussion

We created a cellular-level connectome model of a sensorimotor pathway in larval zebrafish based on known cell types and patterns of synaptic connections. In zebrafish, these interconnected neurons occur in consistent numbers, locations, and with stereotypical connection patterns in virtually all individuals, and form a directional pathway from sensory receptors to motor effectors. Such identifiable neurons are rare in vertebrates, and to the authors' knowledge, this constitutes the largest and most complete identified neuron connectome model described in any vertebrate. The model included information from 15 published studies that focused on separate components of the pathway in zebrafish and included 45 connection rules (Table 1). Most specific connections are still uncharacterized, so the model set these connections stochastically.

We characterized the connectivity of this neural pathway using graph theory, which provides computational strategies for expressing patterns in complex networks. Several recent studies have used graph theory to analyze connectomes at larger spatial scales [17], [18], [30] and the same basic strategies were adapted to analyze this cellular-level connectome. The main findings were that the network has sparse overall connectivity, a broad distribution of degrees (connections per node), and a small-world structure. These properties resulted from the biologically-based connectivity in the zebrafish model because they were not shared by test graphs that were similar but that lacked biological detail. Two other variants of the network that preserved the same biologically-based connections but altered stochastic connection rules yielded very similar results to the connectome model presented here, supporting the conclusions that the biologically-based connectivity was the main determinant of the network's graph properties (File S1).

The zebrafish connectome had a connection density of 2.45%. This connection density results from the biological detail incorporated in the graph. Connection density can vary greatly in neural systems, ranging from 0.1% in sparsely connected networks, to 40% in more densely connected cortical areas [31], but connection densities higher than this have not been reported. In small-world networks overall connection density must be well below100%, because the requirement for high local clustering implies areas of relatively sparse connectivity.

Small-world networks simultaneously maximize high degree of local clustering and short average paths in a network. There are various ways to compute small-worldness, however, by one proposed standard [32], the zebrafish connectome graph had a clear small-world structure. The zebrafish graph included highly interconnected local circuits and direct paths connecting those circuits. For example, the model Mauthner neuron mediated two-step connections between many sensory and spinal nodes. The much lower small-world index of the structured random and random graphs reflects the absence of such specialized nodes. The small-world value for the zebrafish model is consistent with small-worldness reported in other neural systems, including monkey cortex [33], [34], mammalian reticular formation [17], and C. elegans nervous system [34]. In C. elegans, nearly every neurons is identifiable, and small-worldness in this network was also attributable to specific ‘hub’ neurons with above-average number of degrees [35].

Small-world organizations have been observed in other neural connectomes [17], [18] and in non-biological networks such as the internet and social networks, but not in random networks [16]. Several advantages of small-world organization (as compared to random networks) have been demonstrated, including greater stability [36] and more efficient signal propagation [37], [16]. Small-world networks also synchronize to phase oscillator input more readily [16], a property that is relevant to many neural systems. Neural connectivity shows small-worldness across different spatial levels, from connections among individual neurons [35], [38] to connections among entire neocortical regions [33], [39]. Our results add to the evidence that small-worldness is a general principle of neural circuits. The property of small-worldness itself may not distinguish a circuit's information processing functions or capabilities. Examining small-worldness and its factors (clustering coefficient and average path length) within different areas of a network, however, has been linked to functional differences between elements of the network [4], [35].

There was a wide range of degrees in the zebrafish connectome model, and this structural diversity in nodes presumably relates to functional differences among neurons. Nodes with low-degrees, such as neuromasts, likely have simpler functions while highly connected nodes play more complex roles in information processing. There was also a >10 fold range in degrees among the descending neuron group, reflecting large diversity within this population in how different nodes contribute to overall connectivity.

Although much recent research has examined the degree distribution of neural networks [40], [41] it is unclear whether all neural systems can be described with the same degree distribution. There is evidence that on a larger scale (if individual neurons are not considered), or for functionally-defined networks, brain connectivity patterns can be considered scale-free [42]–[44]. Researchers have failed to show scale-free architecture in neural network structure [17], [38], although in some cases, [45] a truncated power-law for degree distribution was found. Despite the lack of clear consensus on whether neural networks are consistently organized in a scale-free manner, computing the degree distribution can provide important information about the existence of hubs, implying subsets of nodes with specialized functions related to their atypical connectivity patterns. It is important to note that although some graphs are both small-world and scale-free, these two measures are independent of each other and contribute separate information about the properties of a connectome.

The high degree of clustering within the zebrafish model suggested the presence of distinct subgroups of nodes, particularly within the descending node population (Table 3). A modularity analysis revealed three subgroups (modules) of descending nodes. These modules arose mainly because the descending nodes within them shared sensory inputs and spinal outputs nodes, not because of direct connections among descending nodes, which accounted for only about 1% of all network connections (Figure 5). While separate modules imply distinct functions for each, no obvious functional distinctions are apparent. The rostral module contains most of the midbrain nMLF and RoM1 nodes, which respond to mechanosensory and visual stimuli [46], [47]. The middle module contains the Mauthner neuron and other escape-related neurons [27] but omits one of the Mauthner series neurons (MiD3cl). Rostral and middle modules each contain diverse morphological types, with widely varying axonal and dendritic morphologies [48] and multiple neurotransmitter phenotypes [24], and there is insufficient information to clearly interpret their grouping by this modularity analysis. The caudal module is comprised of a single anatomical type (cranial relay neurons or t-cells) and is likely to reflect a functional grouping, but the function of these neurons has not been elucidated. An expanded connectome model with other sensory systems projecting to descending nodes and more biological detail would yield different modules and perhaps generate functional hypothesis for biological investigations.

Lesion experiments in neuroscience can provide the best evidence that a certain neural structure performs a certain function. Extremely precise nervous system lesions have been done in zebrafish larvae by focusing a laser on single, identified neurons. Laser ablation of the Mauthner series neurons was shown to increase the latency of escape turns in zebrafish larvae [49]. Presented with an escape eliciting water current stimulus, intact larvae initiated turns in ∼4 ms, but when the Mauthner series is ablated on one side of the brain, latency of escape turns to the opposite side increases to >30 ms. An analogous ablation was performed on the connectome model. Path length from neuromasts to motor neurons served as a proxy for escape latency, with shorter paths equated with faster responses. We only analyzed short paths, with 3 or 4 connections, as paths with greater lengths are less likely to contribute to information processing [31].The Mauthner cell itself is known to mediate 3-step paths between neuromasts and spinal motor neurons in zebrafish larvae, and is alone responsible for the highest speed escape responses to water pulse stimuli directed at the tail [49]. The short latency of behavioral responses to water-current in larvae (∼4 ms) is also consistent with a synaptic path length of 3–4.

We found that path lengths differed depending on where along the body the path originated or ended. This somatotopic organization occurred because brain nodes that received input from different neuromasts had different connectivity to the spinal cord. There are many descending neurons in rostral hindbrain segments with high degrees, and therefore likely to have direct connections to the spinal cord. The caudal hindbrain contained several types of descending neurons, (cranial relay neurons, IC, and CC neurons; Table 2; Figure S3), that projected only a short distance into the rostral spinal cord. Mid-body neuromasts were the most likely to connect to the Mauthner series nodes, which, unlike most descending neurons, connected to all spinal segments. Somatotopic organization has recently been shown within the PLL ganglion [50], but it is not known whether it is maintained in the PLL projection to the hindbrain. The different sensorimotor path lengths associated with different hindbrain segments in the current model offer some initial hypotheses on how PLL somatotopy may be represented at downstream levels of the pathway.

When the 6 nodes representing the Mauthner series were deleted from the model the overall number of short paths decreased. Mauthner series deletion had a much larger effect on 3-step and 4-step paths than deletion of 6 randomly selected nodes from the same segments. This result is consistent with the selective effect of Mauthner series lesion in zebrafish on the latency of escape turns [49]. The node deletions also altered the somatotopic distribution of path lengths. For paths ending in the rostral spinal segments, there was a greater proportional loss of 3-step paths than of 4-step paths, but caudal spinal segments did not show the same vulnerability to loss of 3-step paths. This trend was not predicted, and it is not clear why the rostral spinal cord should be more vulnerable to losing shorter length connections than longer length connections as a result of Mauthner series deletion.

Although our model accurately represents some aspects of the biology, particularly cell types, cell numbers, and elements of connectivity, there were many biological details left out. One of the limitations is the lack of physiological detail. Some physiological information was incorporated, such as the directional tunings of individual hair cells, but overall the model greatly simplifies physiology, for example by not weighting connections and omitting the distinction between inhibitory and excitatory connections. Limitations of analyzing only structural elements of neural connectivity have been widely noted, but graph models of neural connectivity can include physiological detail. For example, chemical and electrotonic synapses were represented as separate networks in a graph model of the C. elegans connectome, and chemical synapses were further distinguished by excitatory or inhibitory postsynaptic effect [35]. Effects of these physiological parameters were measurable as changes in the topological properties of the graph. Future revisions of the current model will include recently published physiological information about the neurotransmitters used by different descending neurons [24].

Despite numerous studies, incomplete information about the structure of this zebrafish connectome still limits the current model. While some connections were hard coded on the basis of biological observations, many others (particularly rules about interneuron connectivity) were assigned stochastically according to general rules because more specific information was not available. Some estimates behind those connectivity rules will almost certainly have to be revised when new observations are reported. Moreover, the structure of the zebrafish larva nervous system is simple only in relative terms, and it is not precisely identical across all individuals. A one-to-one correlation of structural connections in the animal and the model is therefore unlikely to be achieved. Another limitation to the current analysis is that only a subset of a larger sensorimotor connectome is considered. Most of these hindbrain and spinal cord neurons receive input from other sensory systems, so the graph properties described for the PLL pathway cannot be considered properties of the neurons themselves and may not extend to other pathways that share neurons.

Studies of identified neuron connectomes are not new, but have mostly been done in invertebrate systems, where identified neurons are easier to find. At least one mammalian connectome study has taken an identified neuron approach by focusing on the neuromuscular junction where component cells and connections can be precisely defined by their anatomical locations [51]. In contrast, the major investments in vertebrate connectome research, including the Human Connectome Project (an initiative of the National Institutes of Health started in 2009), are focused on connections among brain regions, not individual neurons [52], [53]. This is sensible, as it is still not technically feasible to decode complete mammalian connectomes at the cellular level, but the gap in spatial scales of analysis may mean that identified neuron studies have few points of contact to human connectome research. The fact that graph analysis is an increasingly popular analytic tool in both domains may point the way to integrating connectome research and theory across these spatial levels of analysis.

## Supporting Information

Figure S1 **Fluorescence imaging montage of a 6 day old Brn3c:GFP transgenic zebrafish larvae showing labeling of neuromasts along the body.** Five confocal images takes along the anterior-posterior axis were tiled together in Adobe Photoshop to show the entire body. Posterior neuromasts on the left side of the body are labeled, but other neuromasts of the right PLL can be seen through the transparent body of the larva, as can sensory hair cells of the anterior lateral line and inner ear (not labeled). The number of neuromasts counted in this larva (9) is consistent with counts from published studies [26]. Brn3c:GFP transgenic zebrafish [41], in which a fluorescent protein is expressed in all lateral line neuromasts, was obtained from Herwig Baier at UCSF.
(TIF)

Figure S2 **Estimate of brain interneuron numbers.** The number of brain sensorimotor interneurons was estimated using cell counts from an online atlas of larval zebrafish anatomy (zfin.org). A. Micrograph of a low resolution reference image of one micrograph used for cell counting. The inset shows the area of the larva in the micrograph. Yellow box shows the hindbrain region where descending neurons occur. Total number of methylene blue labeled nuclei within this region was estimated. B A high resolution (>8 megapixels) section of the image in A used to count cells. Green boxes show 3 50 µm sampling areas. C. The results of the cell counts and calculation of total number of cells.
(TIF)

Figure S3 **Co-labeling of a cranial interneuron (pink) and a population of descending neurons in the hindbrain.** This confocal micrograph is a projection of 43 optical section taken 1 µm intervals through the dorsal-ventral axis of the hindbrain in a living 6 day old zebrafish larva. The view is through the dorsal surface of the head. Anterior is up. The dashed line shows the approximate hindbrain-spinal cord boundary. A population of descending neurons was labeled by spinal injection of a fluorescent dye on day 5 (Alexa dextran 647, Sigma; black neurons in panel B) and a single cranial relay neuron was injected with a different color dye (Alexa dextran 488; CRN, pink) on the following day by intrasomal injection. The image was recolored from the original to aid visibility. A. Imaging from the dye channel detecting only the cranial relay neuron. The cell body is located in the caudal most hindbrain segment, and it sends ascending and descending axons to the opposite side of the hindbrain. CRN's are also called ‘t-reticular’ neurons because of their t shape, which is clear in this image. B. The CRN overlaid onto the larger population of labeled descending neurons. One Mauthner neuron is labeled (M). The many hindbrain axon outputs of the CRN can be seen clearly in the context of the other hindbrain descending neurons. Both dye channels were acquired simultaneously. Scale bar = 20 µm.
(TIF)

File S1 **Dedicated and distributed network models of the zebrafish PLL pathway.** The two models are identical to the anatomical model within each compartment (sensory/brain/spinal) but connections between compartments are assigned in either a “distributed” or a “dedicated” fashion. Although the two models differ from the anatomical one in over 20% of the connections, the degree distribution and small-worldness measures for them are very close to our results for the anatomical model.
(DOC)
