# Supplementary figures and images for: Graph Theoretical Model of a Sensorimotor Connectome in Zebrafish
Source: PLoS One. 2012 May 18;7(5):e37292. doi: 10.1371/journal.pone.0037292 (PMC3356276; doi:10.1371/journal.pone.0037292)

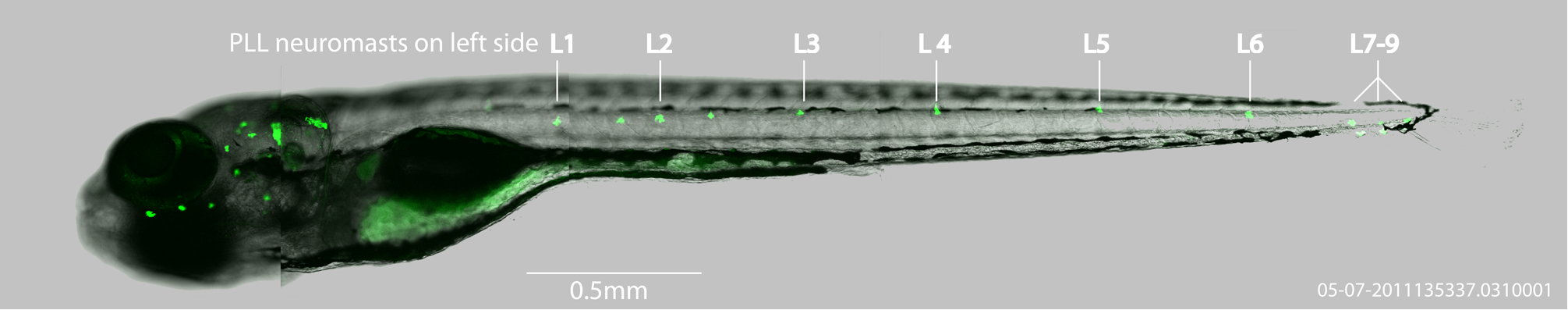

Supplement: Figure S1 — Fluorescence imaging montage of a 6 day old Brn3c:GFP transgenic zebrafish larvae showing labeling of neuromasts along the body. Five confocal images takes along the anterior-posterior axis were tiled together in Adobe Photoshop to show the entire body. Posterior neuromasts on the left side of the body are labeled, but other neuromasts of the right PLL can be seen through the transparent body of the larva, as can sensory hair cells of the anterior lateral line and inner ear (not labeled). The number of neuromasts counted in this larva (9) is consistent with counts from published studies [26]. Brn3c:GFP transgenic zebrafish [41], in which a fluorescent protein is expressed in all lateral line neuromasts, was obtained from Herwig Baier at UCSF. (TIF) [file pone.0037292.s001.tif]

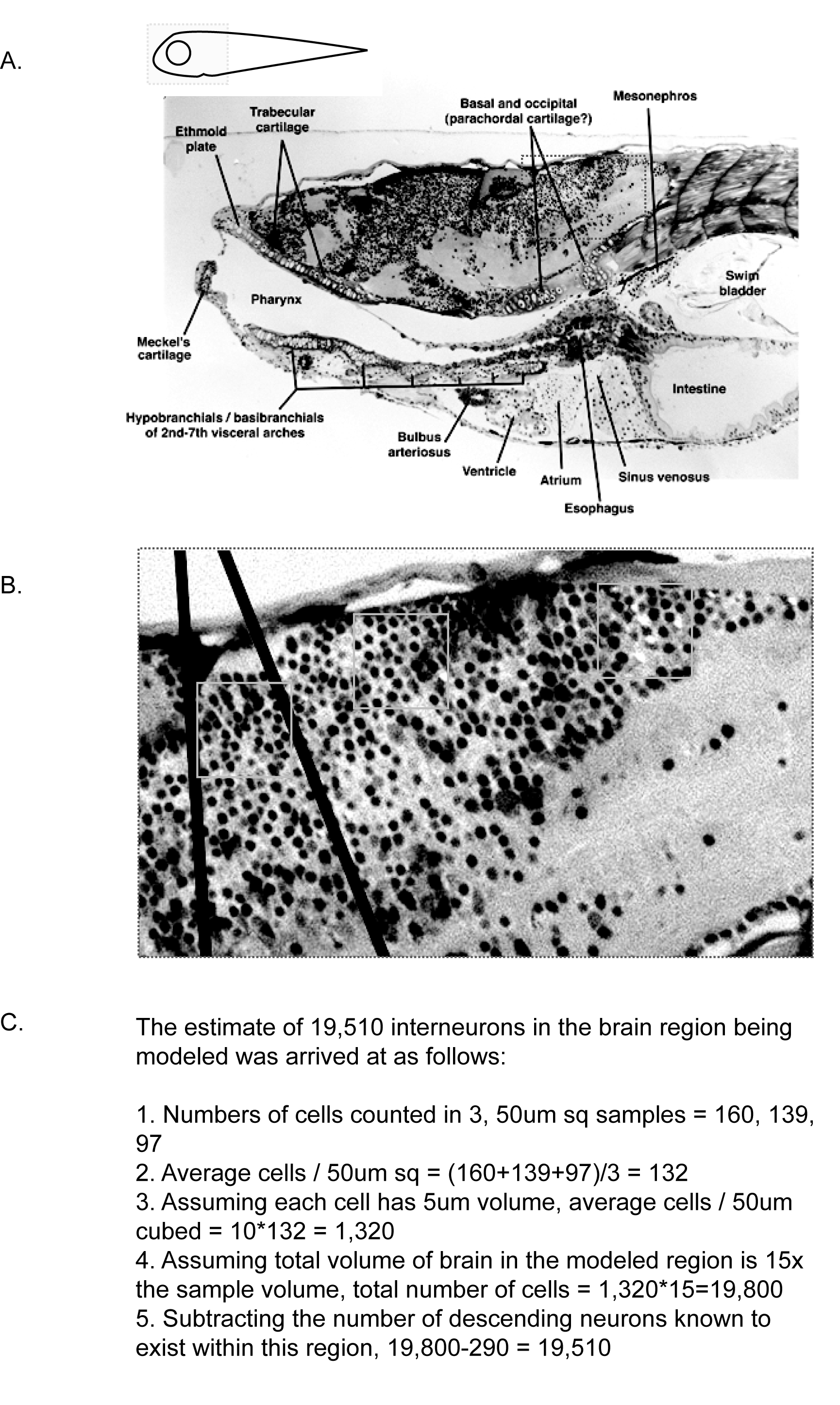

Supplement: Figure S2 — Estimate of brain interneuron numbers. The number of brain sensorimotor interneurons was estimated using cell counts from an online atlas of larval zebrafish anatomy (zfin.org). A. Micrograph of a low resolution reference image of one micrograph used for cell counting. The inset shows the area of the larva in the micrograph. Yellow box shows the hindbrain region where descending neurons occur. Total number of methylene blue labeled nuclei within this region was estimated. B A high resolution (>8 megapixels) section of the image in A used to count cells. Green boxes show 3 50 µm sampling areas. C. The results of the cell counts and calculation of total number of cells. (TIF) [file pone.0037292.s002.tif]

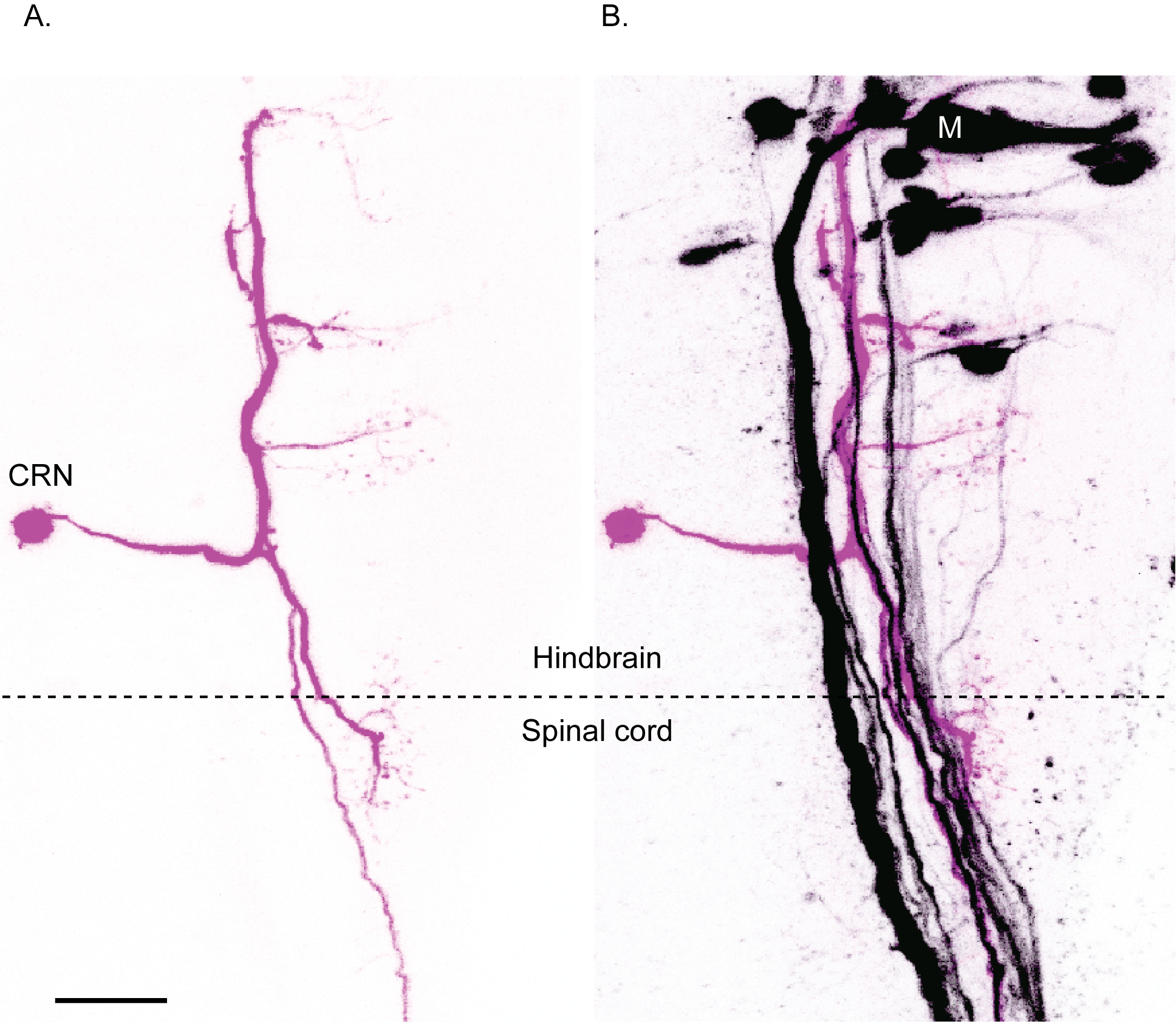

Supplement: Figure S3 — Co-labeling of a cranial interneuron (pink) and a population of descending neurons in the hindbrain. This confocal micrograph is a projection of 43 optical section taken 1 µm intervals through the dorsal-ventral axis of the hindbrain in a living 6 day old zebrafish larva. The view is through the dorsal surface of the head. Anterior is up. The dashed line shows the approximate hindbrain-spinal cord boundary. A population of descending neurons was labeled by spinal injection of a fluorescent dye on day 5 (Alexa dextran 647, Sigma; black neurons in panel B) and a single cranial relay neuron was injected with a different color dye (Alexa dextran 488; CRN, pink) on the following day by intrasomal injection. The image was recolored from the original to aid visibility. A. Imaging from the dye channel detecting only the cranial relay neuron. The cell body is located in the caudal most hindbrain segment, and it sends ascending and descending axons to the opposite side of the hindbrain. CRN's are also called ‘t-reticular’ neurons because of their t shape, which is clear in this image. B. The CRN overlaid onto the larger population of labeled descending neurons. One Mauthner neuron is labeled (M). The many hindbrain axon outputs of the CRN can be seen clearly in the context of the other hindbrain descending neurons. Both dye channels were acquired simultaneously. Scale bar = 20 µm. (TIF) [file pone.0037292.s003.tif]
