# Supplementary material for: Graph Theoretical Model of a Sensorimotor Connectome in Zebrafish
Source: PLoS One. 2012 May 18;7(5):e37292. doi: 10.1371/journal.pone.0037292 (PMC3356276; doi:10.1371/journal.pone.0037292)
Supplement: File S1 — Dedicated and distributed network models of the zebrafish PLL pathway. The two models are identical to the anatomical model within each compartment (sensory/brain/spinal) but connections between compartments are assigned in either a “distributed” or a “dedicated” fashion. Although the two models differ from the anatomical one in over 20% of the connections, the degree distribution and small-worldness measures for them are very close to our results for the anatomical model. (DOC) [file pone.0037292.s004.doc]

**Supporting File S4. Dedicated and distributed network models of the zebrafish PLL sensorimotor pathway**

In our analysis of zebrafish connectivity patterns, we considered two idealized but partially anatomically constrained model networks. The motivation for considering these model networks was to investigate the significance of “dedicated” versus “distributed” connectivity found in certain neural architectures (Morton and Chiel, 1994).

We began the construction of the model networks similar to the anatomical model of the zebrafish and we assigned connections *within* the three compartments as described in Table 1. Our focus was the change in the connectivity patterns *between* these compartments. Thus, the two model networks we created only differed from our anatomical model in the connectivity pattern between the sensory cells and the brain, and the brain and the spine. Our modifications effected about 21% ( 35,800 out of 167,114) of the total network connections. We created both a “dedicated” and a “distributed” connection pattern for the sensory cell-to-brain as well as the brain-to-spine projections. As before, each neuron connected to 120 others.

The distributed pattern between sensory cells and the brain dictated each sensory cell to project to each of the 9 brain regions (7 hindbrain segments, 1 midbrain region and the region containing T, CC and IC cells) represented in our model. The dedicated pattern allows connections from sensory cells to overlapping but distinct bands in the brain. The table, below, organizes the details of this pattern. Note, for example, that sensory neuron 1 projects to regions 1-3 while sensory neuron 5 to regions 2-4. Thus, the projections from these two neurons overlap in some regions but their connections are localized to a few segments of the brain only. Our model aimed to allocate the number of connections in a relatively uniform way, thus (i) most sensory neurons connect to 3 brain regions, with sensory neurons 1,2, 18,19, 20 only connecting to 2 brain regions; (ii) brain regions typically have 5 sensory neurons projecting to them, with a range of at least 4 sensory projections (see region 1) to at most 7 sensory projections (regions 4, 5, 6 and 8).

| Sensory  neurons: | 1,2,3,4 | 1,2,3,4,56 | 3,4,5,6,78 | 5,6,7,8,910,11 | 7,8,9,10,11,12,13 | 9,10,11,12,13,1415 | 12,13,1415,16,  17 | 14,15,1617,18,  19,20 | 16,17,1819,20 |
| --- | --- | --- | --- | --- | --- | --- | --- | --- | --- |
| Brain  region: | 1 | 2 | 3 | 4 | 5 | 6 | 7 | 8 | 9 |

Table S.1.1 Pattern of the localized connections between sensory neurons and brain regions.

Out of the 34,800 projections are made by descending neurons to the spine 18,045 is made to spinal interneurons and 16,755 to motor neurons. In both the dedicated and the distributed models, each descending neuron is assigned the same number of connections to the spine. This simplifying assumption most likely overestimates the significance of certain neuron types, e.g. T-cells while underestimates the role of the Mauthner cell and its homologues. The connection patterns described here are fashioned after the arborization pattern of descending neurons in the spine (Gahtan and O’Malley, 2003). The paper suggests that some RS neurons will have highly localized connections to a few spinal segments only, whereas others may innervate all spinal segments equally.

In the dedicated model case, we assume that 80% of the connections are localized, so projections are made mainly to a few spinal segments and 20% of the projections spread uniformly over the spine. In the distributed model, these percentages are reversed: 80% of projections is spread uniformly to spinal segments and 20% is assigned in localized way. Based on anatomical considerations, the Mauthner cell, its homologues and a few other descending neurons are always counted toward the cells that have uniform connectivity. This group of cells (RoM2l, RoM3m, RoM3l, Mauthner, MiM1, MiV1, MiR1, MiR2, MiV2, MiD2cl, CaD, CaV) always extends projections the whole length (25 segments) of the spine. Overall, 2880 connections are assigned to this group, or 8% of the brain-to-spine connections. Thus, in the dedicated model, 12% of the uniform connections are assigned to descending neurons chosen at random, and in the distributed model, 72%.

Algorithm to generate the uniform connections: The length of projections were set to one of the following: 10, 15, 20, or 25, with probabilities .50, .15, .05, and .30 respectively. Projections always started in the first (most rostral) spinal segment. (For references that support these assumptions, see Table 1, Brain-Motor connections.) The 120 connections for each neuron were then distributed uniformly among the chosen number of spinal segments. Projections from the Mauthner cell and its homologues descend all the way to the last segment (segment 25) of the spine and connect to motor or interneurons in each segment.

Algorithm to generate the localized connections: For each descending neuron, a list of 120 numbers were drawn from a gamma distribution with shape parameter of .5 and rate parameter of 1.8. The resulting numbers were binned into 25 bins, each bin representing one of the 25 spinal segments. The bin numbers gave us the spinal segments to which our descending neuron projects and the number of elements in each bin determined the number of these connections. In order to avoid bias, if the number of connections either exceeded the maximum number of connections allowed in any individual segment or the largest number from the distribution exceeded the maximum number of spinal segments, then the entire list of connections was rejected and another was selected. All connections were assigned starting in the most rostral spinal segment. There was no predetermined projection length for descending neurons with this connectivity, instead, the projection length is a result of the random number drawn from the gamma distribution. The localized connection pattern favors connectivity to rostral spinal segments as small integers (corresponding to spinal segments whose numbering is anterior to posterior) have a larger probability of being chosen from a gamma distribution. This is consistent with anatomical patterns of connectivity from descending neurons that appear denser in rostral than in caudal spinal segments.

We created 100 instantiations of each of the dedicated and the distributed model and included these networks in our test bank. Subsequently, we examined their degree-distribution and the small-worldness. The results of the dedicated and distributed models were very similar to each other and also to the results from our anatomical model. The small-worldness value for the dedicated model was 4.04 while the distributed model was 4.13 (both had standard deviations less than .05). The degree distribution of the dedicated and distributed models is very similar, with small differences reflecting the high degree of connectivity to the rostral spinal segments in the dedicated network (results not shown). Neither model was found scale-free.

References

Morton DW, Chiel HJ. Neural architectures for adaptive behavior. (1994) Trends Neurosci. 17(10):413-20.

Gahtan E, O'Malley DM. Visually guided injection of identified reticulospinal neurons in zebrafish: a survey of spinal arborization patterns.(2003) J Comp Neurol. 28;459(2):186-200.
